# Association of systemic lupus erythematosus autoantibody diversity with breast cancer protection

**DOI:** 10.1186/s13075-021-02449-3

**Published:** 2021-02-25

**Authors:** Ami A. Shah, Takeru Igusa, Daniel Goldman, Jessica Li, Livia Casciola-Rosen, Antony Rosen, Michelle Petri

**Affiliations:** 1 Division of Rheumatology, Johns Hopkins University School of Medicine, 5200 Eastern Avenue, Mason F. Lord Building Center Tower, Suite 4100, Baltimore, MD 21224 USA; 2 Departments of Civil and Systems Engineering, Johns Hopkins University, 3400 North Charles Street, Latrobe Hall 212, Baltimore, MD 21218 USA

**Keywords:** Systemic lupus erythematosus, Cancer, Autoantibodies, Race

## Abstract

**Background:**

Epidemiologic data suggest that patients with systemic lupus erythematosus (SLE) have a lower risk of breast cancer than women in the general population. In light of mechanistic studies suggesting that anti-DNA antibodies have anti-cancer effects, we sought to examine breast cancer risk in autoantibody strata in a well-characterized SLE cohort.

**Methods:**

SLE patients without a cancer diagnosis prior to entry in the Hopkins Lupus Cohort were studied (*N* = 2431). Overall and site-specific cancer incidence was calculated in racial strata and compared with the US Surveillance, Epidemiology and End Results (SEER) registry. Breast cancer incidence was further examined in autoantibody subsets. Patients were considered positive for an autoantibody if they were ever positive for a specificity during their disease course.

**Results:**

Patients with SLE had a 37% lower risk of breast cancer (SIR 0.63, 95% CI 0.39–0.95). The risk of HPV-associated cancers (SIR 4.39, 95% CI 2.87–6.44) and thyroid cancer (SIR 2.27, 95% CI 1.04–4.30) was increased. Cancer risk varied by race, with breast cancer protection occurring in non-African Americans (SIR 0.29, 95% CI 0.11–0.63) and the increased risk of HPV-associated cancers occurring in African Americans (SIR 7.23, 95% CI 4.35–11.3). Breast cancer risk was decreased in patients ever positive for anti-dsDNA (SIR 0.55, 95% CI 0.29–0.96), anti-La (SIR 0.00, 95% CI 0.00–0.78), and lupus anticoagulant (SIR 0.37, 95% CI 0.10–0.94). Patients who were positive for fewer (0–2) SLE autoantibodies did not have a lower risk of breast cancer (SIR 0.84, 95% CI 0.47–1.39), but patients with 3+ autoantibodies had a 59% decreased risk (SIR 0.41, 95% CI 0.16–0.84).

**Conclusions:**

Positivity for multiple SLE autoantibodies was associated with a lower risk of breast cancer, supporting the hypothesis that a highly diversified immune response may exert an anti-cancer effect against some cancers. Validation of racial differences in cancer risk in SLE is required to determine whether cancer screening strategies should be targeted to racial subgroups.

**Supplementary Information:**

The online version contains supplementary material available at 10.1186/s13075-021-02449-3.

## Introduction

Data from several systemic inflammatory rheumatic diseases suggest that distinct immune responses may be markers of increased cancer risk [1]. For example, patients with dermatomyositis and antibodies against transcription intermediary factor 1-gamma (TIF1γ) or nuclear matrix protein 2 (NXP2) have an increased risk of cancer-associated myositis [2], and in systemic sclerosis (scleroderma), patients with anti-RNA polymerase III (POLR3) antibodies have a higher risk of cancer-associated scleroderma [3–5]. Intriguing new data demonstrate that scleroderma patients with anti-centromere antibodies have a lower risk of cancer than that expected in the general population and that scleroderma patients with autoantibodies against both POLR3 and the large subunit of RNA polymerase I (RPA194) have a lower frequency of cancer than those with anti-POLR3 alone [3, 6]. These findings suggest that multiple, orthogonal immune responses targeting linked molecular machinery may confer cancer protection.

In systemic lupus erythematosus (SLE), analyses of multiple large cohorts and international studies have demonstrated a significantly lower risk of breast cancer than that expected in the general population [7–9]. This is intriguing in light of mechanistic studies suggesting that anti-DNA antibodies may have direct anti-cancer effects in cells with DNA repair defects [10–12]. In a landmark study, anti-DNA antibodies were found to be lethal to BRCA2-deficient human cancer cells [11], suggesting that the presence of lupus autoantibodies may contribute to the decreased risk of breast cancer observed in SLE patients. However, it remains unknown whether breast cancer risk in SLE patients varies by autoantibody subtype or with increased diversity of the immune response.

In this study, we examined cancer risk in a large, well phenotyped SLE cohort compared to the general population. We investigated whether breast cancer risk differs by autoantibody subtype and whether the presence of multiple lupus autoantibodies confers greater breast cancer protection.

## Patients and methods

### Study population

Patients seen at the Johns Hopkins Lupus Center for their first visit between 1987 and 2018 were eligible for the study if they consented to participate in the Institutional Review Board (IRB)-approved cohort database and had a diagnosis of SLE by revised American College of Rheumatology (ACR) or Systemic Lupus Collaborating Clinics (SLICC) criteria [13, 14]. The Hopkins Lupus Cohort was approved on a yearly basis by the Johns Hopkins University School of Medicine Institutional Review Board (Study number NA_00039294). Baseline demographic characteristics, including self-identified race, are captured at first visit and categorized as White, Black/African American (AA), Asian, Hispanic, and other. Clinical and serological data were collected prospectively at baseline and quarterly interval visits. SLE characteristics captured included data on SLE manifestations, laboratory tests, treatments, and complications. Data on cigarette smoking, alcohol, and drug consumption were also collected. The following autoantibody categories were examined: dsDNA, RNP, Sm, Ro, La, lupus anticoagulant, anticardiolipin, and beta 2 glycoprotein 1. Autoantibodies were assayed in the same lab as part of routine clinical care: anti-dsDNA by Crithidia; anti-RNP, Sm, Ro, and La by chemiluminescence immunoassay; lupus anticoagulant by testing of Russel Viper Venom Time (RVVT) with confirmatory mixing studies; and anticardiolipin (ACL) and beta 2 glycoprotein 1 (including IgG, IgM, and IgA) by ELISA. Patients were considered positive for an autoantibody if they were ever positive during their disease course, regardless of whether they were positive or negative for other autoantibodies. Because autoantibody overlap is common in SLE, we also examined patients in two groups: those who were positive for 0–2 versus 3 or more autoantibodies. The timing of SLE onset was defined by the first SLE manifestation by ACR or SLICC criteria. Patient-reported cancer diagnoses and dates of diagnosis were confirmed by review of pathology and/or oncology notes.

### Examination of cancer risk in SLE compared with the general population

Demographic, clinical, and laboratory characteristics were compared between patients with a history of cancer after SLE onset and SLE patients without a cancer diagnosis. Continuous variables were assessed by an independent two-sample t-test with pooled variance and dichotomous/categorical variables were compared by chi-square tests.

Cancer risk was calculated by comparing cancer incidence in the Hopkins Lupus Cohort with the Surveillance, Epidemiology and End Results (SEER) registry, which is a nationally representative sample of the US population. Standardized incidence ratios (SIRs) were calculated for cancer overall and individual cancer types. Human papillomavirus (HPV)-associated cancers, including cancers of the cervix, vagina/vulva, and anus, were also examined as a group given prior data suggesting a higher risk of virus-associated cancers in SLE. The observed number of cancers in our cohort was compared with the expected number of cancer cases for the US population by identifying the crude rate of incident cancers corresponding to each patient’s age (within 5-year intervals), sex, race, ethnicity, and the calendar year of exposure in SEER [15]. Due to the long duration of the cohort, the SEER 9 Registries Research Data was chosen for our US comparison dataset. Person time prior to 1973 was not examined as this dataset begins in 1973. At the time of analysis, SEER data were complete through 2014. SEER crude rates for 2014 were used as a surrogate for person time after 2014. The sum of the crude rates for all years of exposure for all patients yielded the expected number of cancer cases. We used the three SEER data race designations (White, Black, and other) to account for race in our expected number calculations. To find the 95% confidence limits, we used the expressions for the exact confidence limits for the true SIR as previously described [15–17]. The procedure in [18] was used to compute *p* values. These formulas, which correspond to Fisher’s exact test, have been shown to be conservative, requiring more evidence than is necessary to reject a false null hypothesis. While the less conservative mid *P* tests have been advocated by statisticians [19], we have used Fisher’s exact test because we believe it has been most widely used in SEER-based SIR studies.

Cancer risk was assessed after entry into the Hopkins Lupus Cohort. Patients with cancers preceding this time window were excluded from our analysis. Administrative censoring occurred at the cancer diagnosis date or last visit date, whichever came first. The study population for our primary analyses comprised 2431 SLE patients.

As our analyses demonstrated that patients with SLE had differential cancer risk by race, cancer incidence data are presented for the overall SLE cohort and in racial strata (African American (AA); non-AA, which includes White, White-Hispanic, Asian and other). Stratified analyses present risk estimates in AA SLE patients compared to AAs in the general population; similarly, risk estimates for non-AA SLE patients are relative to non-AAs in the general population. Given the striking extremes of cancer risk in SLE—that is the markedly lower risk of breast cancer and significantly higher risk of HPV-associated cancers—we graphically examined the age-adjusted incidence of these tumor types in patients with SLE, stratified by race, compared to the general population. Lastly, because we hypothesized that anti-dsDNA antibodies and the presence of multiple immune responses may confer protection for breast cancer in SLE, we further examined the risk of breast cancer in SLE patients in distinct autoantibody strata and by whether patients were positive for few (0–2) or many (3+) autoantibodies.

Analyses were performed using MATLAB R2016b (MathWorks, Natick, Massachusetts, USA) and R V.3.4.0 (R Foundation, Vienna, Austria) to verify SIR calculations. *P* values ≤ 0.05 were considered to be statistically significant; when appropriate, Bonferroni adjustment for multiple comparisons was performed, as noted in the tables.

## Results

Among the 2475 patients enrolled in the Hopkins Lupus Cohort, 163 (6.6%) had a history of cancer, occurring at a mean duration of 15.61 ± 9.80 years after SLE onset. Of these 163 cancer cases, 44 cancers occurred prior to entry into our Lupus Cohort, and these patients were not included in our study population. Demographic, social and clinical characteristics of the remaining 119 SLE patients with cancer compared to 2312 SLE patients without a history of cancer are shown in Table 1 and Supplemental Tables 1–3. SLE patients with a history of cancer had an older age at SLE onset (32.9 ± 12.3 vs. 28.3 ± 12.5 years, *p* < 0.001) and at cohort entry (41.2 ± 13.6 vs. 36.6 ± 12.5 years, *p* < 0.001), and a longer follow-up duration (13.2 ± 7.9 vs. 6.9 ± 7.1 years, *p* < 0.001), than those without cancer. SLE patients with a history of cancer were also more likely to be African-American (AA) (54.6% vs. 39.0%, *p* < 0.001) and have ever smoked (45.4% vs. 35.6%, *p* = 0.03). After adjusting for multiple comparisons, SLE patients with cancer were more likely to have thrombocytopenia (31.9% vs. 19.5%, *p* < 0.001), pulmonary fibrosis (19.3% vs. 8.7%, *p* < 0.001), and vasculitis (25.2% vs. 13.4%, *p* < 0.001) as features of their SLE (Supplemental Table 1). Those with cancer were less likely to be anti-La (6.7% vs. 13.2%, *p* = 0.039) positive, but more likely to have anticardiolipin (60.5% vs. 45.2%, *p* = 0.001) antibodies (Supplemental Table 2). There were no differences in exposure to various immunomodulatory therapies, including hydroxychloroquine, mycophenolate mofetil, azathioprine, cyclophosphamide, belimumab, rituximab, and prednisone (Supplemental Table 3). However, those with cancer were more likely to have ever been on hormone replacement therapy (21.8% vs. 13.2%, *p* = 0.008).

**Table 1:** Demographic and social characteristics of the Hopkins Lupus Cohort by history of cancer

Characteristic	Overall (*N* = 2431)	Cancer (*N* = 119)	No cancer (*N* = 2312)	*p* value*
Age of SLE onset, mean (SD)	28.5 (12.5)	32.9 (12.3)	28.3 (12.5)	< 0.001
Age at cohort entry, mean (SD)	36.8 (12.6)	41.2 (13.6)	36.6 (12.5)	< 0.001
First visit to last visit (years, mean (SD))	7.2 (7.3)	13.2 (7.9)	6.9 (7.1)	< 0.001
Female, no. (%)	2243 (92.3)	106 (89.1)	2137 (92.4)	0.19
Race, no. (%)				
White	1271 (52.3)	53 (44.5)	1218 (52.7)	0.08
Black/African American	966 (39.7)	65 (54.6)	901 (39.0)	< 0.001
Other**	194 (8.0)	1 (0.8)	193 (8.3)	0.003
Smoking status (ever smoker), no. (%)	877 (36.1)	54 (45.4)	823 (35.6)	0.030
Alcohol (past)	167 (6.9)	13 (10.9)	154 (6.7)	0.08
Cancer-SLE interval, years (mean (SD))	8.24 (6.70)	8.24 (6.70)		
Deceased, no. (%)	194 (8.0)	25 (21.0)	169 (7.3)	< 0.001

*p value for comparison between cancer and no cancer groups

**Asian (*N* = 100, 4.1%), not Asian, White, Black/African-American or unspecified (*N* = 94, 3.9%)

Among the 119 patients with SLE and cancer, the most common cancer type was breast, which occurred in 22 patients. This was followed by vagina/vulva (13), cervical (11), lung (11), hematologic (10), colorectal (10), and thyroid (9). A full list of all cancer types observed is provided in Supplemental Table 4.

### Cancer risk and type varies in racial subgroups in SLE compared to the general population

Patients with SLE did not have an increased risk of cancer compared to age, sex, race, ethnicity, and calendar time-matched general population controls (SIR 1.16, 95% CI 0.96–1.39; Table 2). However, patients with SLE had a 37% lower risk of breast cancer (SIR 0.63, 95% CI 0.39–0.95). At the other extreme, patients with SLE had a markedly increased risk of HPV-associated cancers (SIR 4.39, 95% CI 2.87–6.44), particularly cancers of the cervix (SIR 2.73, 95% CI 1.36–4.89) and vagina/vulva (SIR 6.87, 95% CI 3.66–11.8). An increased risk of thyroid cancer was also noted (SIR 2.27, 95% CI 1.04–4.30).

**Table 2:** Risk of cancer after SLE cohort entry compared to general population controls (*N* = 2431 SLE patients contributing 20,399 person-years)^#^

Site	No. obs.	No. exp.	SLE cohort SIR (95%CI)	***p*** value	AA* SIR (95%CI)	AA ***p*** value	Non-AA^**^**^ SIR (95%CI)	Non-AA ***p*** value
All	119	102.3	1.16 (0.96–1.39)	0.12	1.63 (1.26–2.08)	**< 0.001**	0.86 (0.65–1.13)	0.31
**Female cancers**								
Breast	22	34.9	0.63 (0.39–0.95)	**0.027**	1.13 (0.65–1.84)	0.69	0.29 (0.11–0.63)	**< 0.001**
Cervix	11	4	2.73 (1.36–4.89)	**0.006**	3.78 (1.52–7.79)	**0.006**	1.84 (0.50–4.71)	0.35
Vagina/vulva	13	1.9	6.87 (3.66–11.8)	**< 0.001**	14.13 (7.05–25.3)	**< 0.001**	1.80 (0.22–6.49)	0.61
**Other cancers**								
Hematologic	10	6.5	1.54 (0.74–2.84)	0.24	1.87 (0.61–4.36)	0.27	1.31 (0.43–3.07)	0.67
Lung	11	8.7	1.27 (0.63–2.26)	0.52	1.84 (0.74–3.80)	0.18	0.82 (0.22–2.09)	0.92
Colorectal	10	7.2	1.39 (0.66–2.55)	0.38	1.18 (0.32–3.01)	0.88	1.57 (0.58–3.42)	0.37
Thyroid	9	4	2.27 (1.04–4.30)	**0.041**	0.00 (0–3.31)	0.66	3.15 (1.44–5.98)	**0.006**
Melanoma	8	5.4	1.48 (0.64–2.91)	0.36	0.00 (0–37.3)	0.99	1.51 (0.65–2.97)	0.34
HPV-associated cancers**	26	5.9	4.39 (2.87–6.44)	**< 0.001**	7.23 (4.35–11.3)	**< 0.001**	2.13 (0.86–4.38)	0.10

Data for cancer sites with < 5 cases observed after cohort entry are not presented, including the uterus, ovary, fallopian tube, prostate, multiple myeloma, salivary gland, stomach, duodenal and other small bowel, anus, pancreas, liver, kidney, bladder, brain, and unknown primary

**HPV-associated cancers include cervix, vulva/vagina, and anus cancers

AA = African American; non-AA = non-African American

*N = 966 SLE patients contributing 8488 person-years

^^^*N* = 1465 SLE patients contributing 11,911 person-years

^#^When adjusting for multiple comparisons (10 per racial strata), *p* ≤ 0.005 is considered statistically significant

The higher or lower risk of these different tumor types varied in racial subgroups (Table 2). Non-AAs with SLE had a striking 71% lower risk of breast cancer compared to non-AAs in the general population (SIR 0.29, 95% CI 0.11–0.63). In contrast, AAs with SLE were not protected from breast cancer compared to AAs in the general population (SIR 1.13, 95% CI 0.65–1.84). The high risk of HPV-associated cancers in SLE is primarily due to a markedly increased risk of these cancers in AAs with SLE compared to AAs in the general population (SIR 7.23, 95% CI 4.35–11.3). In contrast, the risk of HPV-associated cancers in non-AA with SLE was not statistically significantly higher than that expected in non-AA in the general population (SIR 2.13, 95% CI 0.86–4.38). Lastly, the increased risk of thyroid cancer was only seen in the non-AA subset of SLE patients (SIR 3.15, 95% CI 1.44–5.98).

**Fig. 1:**
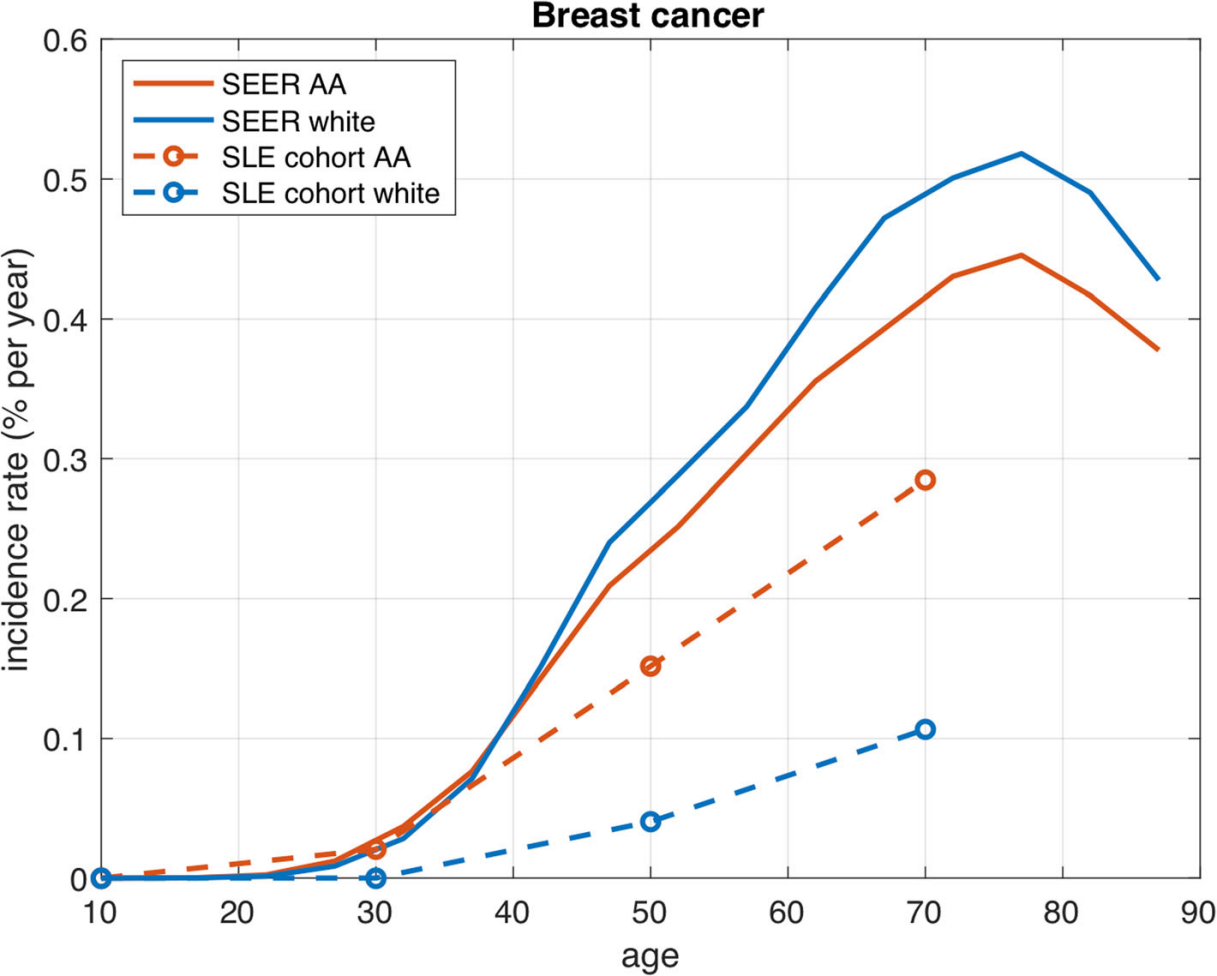
Breast cancer incidence rates in racial strata in the SEER registry and the Johns Hopkins Lupus Center cohort. Breast cancer incidence is lower in SLE patients than that expected in the general population, particularly for patients of white race. SEER, Surveillance; Epidemiology and End Results (SEER) registry; AA, African American

In exploratory analyses, we examined the age-adjusted incidence rate of breast cancer graphically in racial strata, comparing patients with SLE to the general population (Fig. 1). After the age of 40, AA women in the general population have a lower incidence rate of breast cancer than white women. In contrast, for patients with SLE, who generally have a lower incidence rate than that seen in the overall population, this incidence rate is higher and rises faster for AA women than white women.

**Fig. 2:**
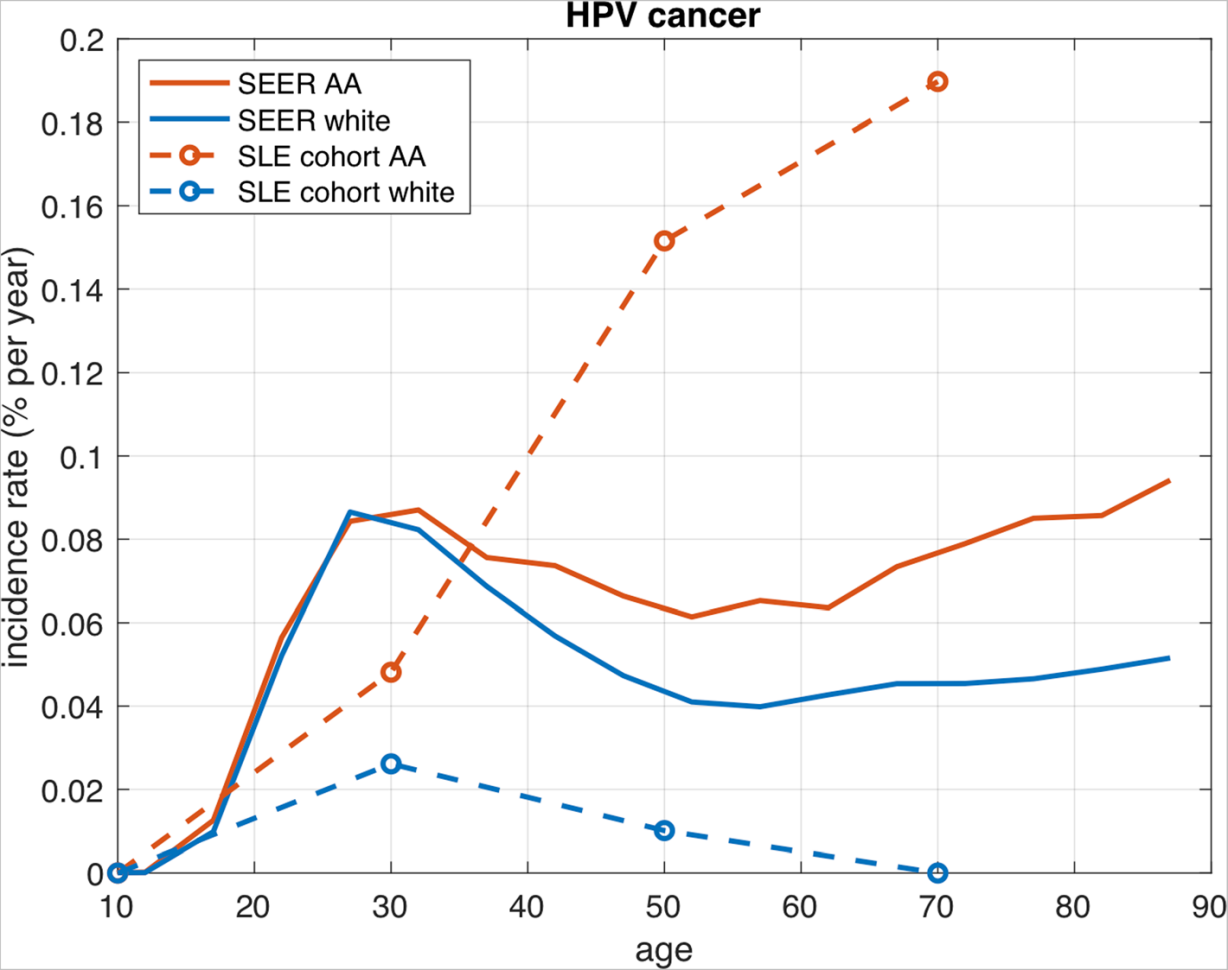
Cancer incidence rates for HPV-associated cancers across racial strata in the SEER registry and the Johns Hopkins Lupus Center cohort. HPV-associated cancers include cancers of the cervix, vagina/vulva, and anus. HPV-associated cancer incidence is substantially higher in African American women with SLE, compared to that expected in African American women in the general population. SEER, Surveillance, Epidemiology and End Results (SEER) registry; AA, African American

For HPV-associated cancers, the incidence rate is higher among AA women in the general population than their white counterparts after approximately the age of 30 (Fig. 2). The age-adjusted incidence rate is dramatically higher and increases steeply in AA women with SLE.

### Breast cancer risk is decreased in distinct SLE autoantibody subsets, with positivity for more autoantibodies conferring increased protection in non-AA patients

It remains unknown why patients with SLE have a significantly lower risk of breast cancer than that expected in the general population. Given recent mechanistic data demonstrating that anti-DNA antibodies have direct anti-cancer effects in cells with DNA repair defects [11], we were especially interested in investigating breast cancer risk in SLE autoantibody subsets. Patients with anti-dsDNA (SIR 0.55, 95% CI 0.29–0.96), La (SIR 0.00, 95% CI 0.00–0.78) and lupus anticoagulant (SIR 0.37, 95% CI 0.10–0.94) had a decreased risk of breast cancer (Table 3). As patients with SLE are often positive for multiple autoantibodies, we investigated whether the presence of several different antibody specificities conferred greater cancer protection. This analysis showed that SLE patients with 0–2 autoantibody specificities did not have a decreased risk of breast cancer. In contrast, patients positive for 3 or more different autoantibodies had a 59% decreased risk (Table 4; SIR 0.41, 95% CI 0.16–0.84).

**Table 3:** Breast cancer risk after cohort entry in distinct autoantibody strata, compared to the general population^#^

Antibody	Sample size	No. obs.	No. exp.	SLE cohort SIR (95% CI)	***p*** value	AA* SIR (95% CI)	AA ***p*** value	Non-AA^**^**^ SIR (95% CI)	Non-AA ***p*** value
All	2431	22	34.9	0.63 (0.39–0.95)	**0.027**	1.13 (0.65–1.84)	0.69	0.29 (0.11–0.63)	**< 0.001**
dsDNA	1503	12	21.7	0.55 (0.29–0.96)	**0.035**	0.97 (0.44–1.84)	0.99	0.24 (0.05–0.71)	**0.003**
RNP	709	9	7.3	1.24 (0.56–2.34)	0.62	1.78 (0.77–3.50)	0.17	0.36 (0.01–2.00)	0.47
Sm	507	2	4.9	0.41 (0.05–1.47)	0.27	0.34 (0.01–1.90)	0.42	0.51 (0.01–2.83)	0.83
Ro	756	4	10.2	0.39 (0.11–1.01)	0.053	0.23 (0.01–1.26)	0.13	0.52 (0.11–1.52)	0.35
La	313	0	4.7	0.00 (0.00–0.78)	**0.018**	0.00 (0.00–2.28)	0.40	0.00 (0.00–1.19)	0.09
LAC	615	4	11	0.37 (0.10–0.94)	**0.031**	0.92 (0.25–2.36)	0.99	0.00 (0.00–0.56)	**0.003**
ACL	1118	14	19.9	0.70 (0.38–1.18)	0.21	1.35 (0.67–2.42)	0.40	0.25 (0.05–0.74)	**0.005**
Beta2GP1	446	4	9	0.44 (0.12–1.13)	0.11	0.60 (0.07–2.17)	0.71	0.35 (0.04–1.27)	0.15

^#^When adjusting for multiple comparisons (9 per racial strata), *p* ≤ 0.006 is considered statistically significant

*LAC* lupus anticoagulant, *ACL* anticardiolipin, *Beta2GP1* beta 2 glycoprotein 1, *AA* African American; *non-AA* non-African American

*N = 966 SLE patients contributing 8488 person-years

^^^*N* = 1465 SLE patients contributing 11,911 person-years

**Table 4:** Breast cancer risk as a function of number of SLE autoantibodies, compared to the general population

Number of autoantibodies	N	No. obs.	No. exp.	SLE cohort SIR (95%CI)	***p*** value	AA* SIR (95%CI)	AA ***p*** value	Non-AA^ SIR (95%CI)	Non-AA ***p*** value
0–2 antibodies	1301	15	17.8	0.84 (0.47–1.39)	0.60	1.72 (0.86–3.08)	0.12	0.35 (0.10–0.90)	**0.023**
3+ antibodies	1130	7	17.1	0.41 (0.16–0.84)	**0.010**	0.65 (0.21–1.51)	0.43	0.21 (0.03–0.77)	**0.010**

*N* sample size, *AA* African American, *non-AA* non-African American

*N = 966 SLE patients contributing 8488 person-years

^*N* = 1465 SLE patients contributing 11,911 person-years

As our initial analyses demonstrated that AAs with SLE were not protected from breast cancer, we further examined the association between breast cancer risk and autoantibody type and number in racial strata. It is important to note, however, that our statistical power is limited for individual autoantibodies when stratifying by race. Non-AA patients with SLE had a lower risk of breast cancer if they were positive for anti-dsDNA (SIR 0.24, 95% CI 0.05–0.71), lupus anticoagulant (SIR 0.00, 95% CI 0.00–0.56), or ACL (SIR 0.25, 95% CI 0.05–0.74) (Table 3), and the presence of more autoantibodies was associated with a lower point estimate for breast cancer risk (Table 4). Among AAs, however, this dose response finding was attenuated and was not statistically significant.

## Discussion

It has long been appreciated that patients with SLE have an increase in cancer risk compared to individuals in the general population, with data suggesting a higher risk of hematologic, lung, thyroid, liver, bladder, pancreatic, kidney, nasopharyngeal and HPV-associated (vaginal, vulvar, cervical, anal) malignancies and a lower risk of breast, endometrial, and possibly ovarian and prostate cancers [7–9, 20–23]. The decreased risk of breast cancer in SLE patients has been intriguing in light of mechanistic data suggesting that cell-penetrating dsDNA antibodies may have anti-cancer effects in cells with DNA repair defects, such as BRCA2-deficient human cancer cells [11]. This, combined with recent data in scleroderma suggesting that unique immune responses and combinations of autoantibodies may associate with decreased cancer risk [3, 6], raises the question as to whether dsDNA autoantibodies and/or the presence of multiple autoantibodies confers breast cancer protection in SLE.

In this study, we utilized the large, well-phenotyped Hopkins Lupus cohort to examine cancer risk in lupus patients compared to the general population, adjusting our risk estimates by age, sex, race, ethnicity, and calendar time. Our data confirm a higher risk of HPV-associated malignancies and thyroid cancer and a lower risk of breast cancer in SLE. To our knowledge, our data demonstrate for the first time that there are striking racial differences in cancer risk and type among SLE patients—notably, AA patients with SLE had a markedly elevated risk of HPV-associated cancers, and non-AA with SLE had a 71% decreased risk in breast cancer. Patients with anti-dsDNA, anti-La, and lupus anticoagulant had a statistically significantly lower breast cancer risk. The presence of 3 or more SLE autoantibodies was associated with a significantly lower risk of breast cancer, a novel finding that was further strengthened among non-AA patients with SLE. These data provide additional support for the hypothesis that a highly diversified immune response may have a cancer-protective effect in the rheumatic diseases.

Few studies have investigated risk factors for breast cancer risk or protection in SLE. In a case-cohort analysis with 86 SLE breast cancer cases and 4498 female SLE cancer-free controls, only older age was associated with breast cancer risk in adjusted analyses [24]. There was no decrease in breast cancer risk observed with anti-dsDNA positivity, which was modeled as a weighted average of the number of times patients were anti-dsDNA positive over time. Of note, dsDNA autoantibodies were assayed in multiple different centers using a variety of assay methodologies. Anti-dsDNA titers and the presence of multiple SLE immune responses were not examined. Anti-malarial drugs were not shown to be breast cancer protective in this study, in contrast to some earlier studies [25, 26].

Only a few studies have examined racial differences in breast cancer risk in SLE patients, with conflicting results. One study, which examined Medicare claims data and therefore was skewed to an older population, found that breast cancer risk in Caucasian and AA SLE patients was similar to that expected in these racial subgroups in the general population [27]. Another study utilizing the California Patient Discharge Dataset identified a lower risk of breast cancer in SLE patients, but this was only statistically significant among non-Hispanic whites [28]. In a lupus cohort from Cook County, Illinois, the risk of breast cancer was increased only in Caucasian women [29]. In a multicenter, international cohort of SLE patients, the SIR for breast cancer was decreased in black/AA SLE patients but not in white SLE patients, which is in contrast to our findings [30]. Of note, that study, which included patients from North America, the UK, Europe, and Asia, used the US-based SEER registry as a comparator group to estimate the SIR, which may affect the validity of these estimates. Our data suggest that further study is required to determine if there are racial differences in breast cancer risk in SLE, and to explore whether these differences could be due to traditional breast cancer risk factors, genetic framework, SLE activity/severity, use of immunomodulatory therapy, and types/combinations of immune responses, among other factors.

Prior studies have suggested that the decrease in breast cancer risk observed in SLE may be due to a lower risk of hormone receptor-negative tumors [31]. It has been hypothesized that triple-negative breast cancers, which are known to harbor defects in DNA repair, may be more susceptible to cell-penetrating anti-DNA antibodies [32]. An important limitation of our study was our relatively small number of breast cancer cases, which precluded our ability to examine specific breast cancer subtypes.

While not the primary focus of our study, we confirmed that there is an increased risk for HPV-associated cancers in SLE, and to our knowledge noted for the first time that this risk increase is seen primarily in AA patients with SLE. Many studies have investigated the risk of and risk factors for cervical intraepithelial neoplasia (CIN) and cervical cancer in SLE [33–35]. The data suggest that the risk is increased with immunosuppression [33], particularly with cyclophosphamide [35]. It has been hypothesized that persistent infection with oncogenic HPV subtypes may develop in the context of inflammation or immunosuppression, predisposing SLE patients to CIN or cervical cancer. Further work is needed to determine whether the racial differences observed in our study are due to potential confounders such as disease activity, immunosuppression, infection with different high-risk HPV genotypes (including those not covered by current HPV vaccines) [36], or traditional cervical cancer risk factors.

Our study has important limitations requiring additional research probing the cancer-autoimmunity connection in SLE. Further study in a larger sample size is needed to investigate specific combinations of SLE immune responses that may be breast cancer protective, and mechanistic studies are required to determine if these autoantibodies, alone or in combination, exert an anti-cancer effect. In addition, it will be important to define whether there are interactions between race, genetic framework and autoantibody type. Work is also needed to define whether breast cancer risk varies by anti-DNA antibody titer and subtype over time as there may be differential cancer risk by strength of the immune response and in patients with cell-penetrating anti-DNA antibodies versus other anti-DNA antibodies. Our study was also underpowered to assess whether the decrease in breast cancer risk observed in autoantibody subgroups was due to a decrease in specific breast cancer subtypes, such as hormone receptor-positive versus triple-negative tumors.

## Conclusions

Our data suggest there may be differential cancer risk and type across racial groups in SLE, and that a greater breadth of the SLE immune response as assessed by autoantibodies may associate with breast cancer protection particularly in non-AA patients. Further study is required to validate these findings and to determine whether cancer screening strategies should be targeted to distinct racial and autoantibody subgroups. Investigating the mechanistic basis for differences in cancer risk in these subgroups has the potential to improve our understanding of SLE autoimmunity and anti-tumor immunity.

## Supplementary Information

**Additional file 1: Supplemental Table 1.** SLE characteristics by cancer status. **Supplemental Table 2.** Laboratory parameters and serologies by cancer status. **Supplemental Table 3.** Drug exposure (ever use) by cancer status. **Supplemental Table 4.** Cancer sites observed in the Hopkins Lupus Cohort (*N* = 119 cancer cases).

## Data Availability

Data are available upon reasonable request by contacting Michelle Petri, MD MPH (mpetri@jhmi.edu).

## Abbreviations

AA: African Americans
ACL: Anticardiolipin antibody
ACR: American College of Rheumatology
BRCA2: BReast CAncer gene 2
CIN: Cervical intraepithelial neoplasia
HPV: Human papillomavirus
IRB: Institutional Review Board
NXP2: Nuclear matrix protein 2
POLR3: RNA polymerase III
RPA194: Large subunit of RNA polymerase I
SEER: Surveillance, Epidemiology and End Results registry
SIR: Standardized incidence ratio
SLE: Systemic lupus erythematosus
SLICC: Systemic Lupus Collaborating Clinics
TIF1g: Transcription intermediary factor 1-gamma
