# Supplementary material for: Association of systemic lupus erythematosus autoantibody diversity with breast cancer protection
Source: Arthritis Res Ther. 2021 Feb 25;23:64. doi: 10.1186/s13075-021-02449-3 (PMC7905617; doi:10.1186/s13075-021-02449-3)
Supplement: Supplementary file 1 — Additional file 1: Supplemental Table 1. SLE characteristics by cancer status. Supplemental Table 2. Laboratory parameters and serologies by cancer status. Supplemental Table 3. Drug exposure (ever use) by cancer status. Supplemental Table 4. Cancer sites observed in the Hopkins Lupus Cohort (N = 119 cancer cases). [file 13075_2021_2449_MOESM1_ESM.docx]

**Supplementary Tables.**

**Supplemental Table 1. SLE characteristics by cancer status.**

| **Characteristic** |  | |  | **Overall (N=2431)** | **Cancer (N=119)** | **No cancer (N=2312)** | **p-value*** |
| --- | --- | --- | --- | --- | --- | --- | --- |
| Fever |  | |  | 850 (35.0) | 52 (43.7) | 798 (34.5) | 0.040 |
| Lymphadenopathy | | |  | 768 (31.6) | 45 (37.8) | 723 (31.3) | 0.14 |
| Splenomegaly |  | |  | 102 (4.2) | 4 (3.4) | 98 (4.2) | 0.67 |
| Hematologic |  | |  |  |  |  |  |
|  | Lymphopenia | |  | 1008 (41.5) | 63 (52.9) | 945 (40.9) | 0.010 |
|  | Leukopenia | |  | 1134 (46.6) | 65 (54.6) | 1069 (46.2) | 0.07 |
|  | Anemia | |  | 1527 (62.8) | 89 (74.8) | 1438 (62.2) | 0.006 |
|  | Hemolytic anemia | |  | 234 (9.6) | 13 (10.9) | 221 (9.6) | 0.64 |
|  | Coombs positivity | |  | 421 (17.3) | 26 (21.8) | 395 (17.1) | 0.19 |
|  | Thrombocytopenia | |  | 488 (20.1) | 38 (31.9) | 450 (19.5) | <0.001 |
| Cutaneous |  | |  |  |  |  |  |
|  | Malar | |  | 1179 (48.5) | 60 (50.4) | 1119 (48.4) | 0.67 |
|  | Discoid | |  | 474 (19.5) | 34 (28.6) | 440 (19.0) | 0.010 |
|  | Photosensitivity | |  | 1253 (51.5) | 71 (59.7) | 1182 (51.1) | 0.07 |
|  | Mucosal ulcers | |  | 1263 (52.0) | 72 (60.5) | 1191 (51.5) | 0.06 |
|  | Alopecia | |  | 1361 (56.0) | 74 (62.2) | 1287 (55.7) | 0.16 |
| Cardiopulmonary | |  |  |  |  |  |  |
|  | Pleuritis | |  | 1047 (43.1) | 53 (44.5) | 994 (43.0) | 0.75 |
|  | Pulmonary fibrosis | |  | 224 (9.2) | 23 (19.3) | 201 (8.7) | <0.001 |
|  | Pericarditis | |  | 531 (21.8) | 33 (27.7) | 498 (21.5) | 0.11 |
|  | Myocarditis | |  | 43 (1.8) | 5 (4.2) | 38 (1.6) | 0.034 |
|  | Pulmonary hypertension | |  | 192 (7.9) | 16 (13.4) | 176 (7.6) | 0.022 |
| Gastrointestinal lupus | |  |  | 116 (4.8) | 10 (8.4) | 106 (4.6) | 0.06 |
| Renal |  | |  |  |  |  |  |
|  | Hematuria | |  | 681 (28.0) | 37 (31.1) | 644 (27.9) | 0.45 |
|  | Proteinuria | |  | 1089 (44.8) | 55 (46.2) | 1034 (44.7) | 0.75 |
| Neurologic features |  | |  | 1103 (45.4) | 59 (49.6) | 1044 (45.2) | 0.35 |
| Myositis |  | |  | 178 (7.3) | 13 (10.9) | 165 (7.1) | 0.12 |
| Vasculitis |  | |  | 340 (14.0) | 30 (25.2) | 310 (13.4) | <0.001 |
| Livedo |  | |  | 636 (26.2) | 30 (25.2) | 606 (26.2) | 0.81 |
| Thrombotic APS |  | |  | 368 (15.1) | 28 (23.5) | 340 (14.7) | 0.009 |

All values represented are numbers (percentage), unless otherwise specified.

*p-value for comparison between cancer and no cancer groups. After adjusting for multiple comparisons, p≤0.002 is considered statistically significant.

**Supplemental Table 2. Laboratory parameters and serologies by cancer status.**

| **Laboratory parameter** |  | **Overall (N=2431)** | **Cancer (N=119)** | **No cancer (N=2312)** | **p-value*** |
| --- | --- | --- | --- | --- | --- |
| Low C3 |  | 1344 (55.3) | 71 (59.7) | 1273 (55.1) | 0.32 |
| Low C4 |  | 1158 (47.6) | 61 (51.3) | 1097 (47.4) | 0.41 |
| dsDNA |  | 1503 (61.8) | 81 (68.1) | 1422 (61.5) | 0.15 |
| RNP |  | 709 (29.2) | 38 (31.9) | 671 (29.0) | 0.50 |
| Sm |  | 507 (20.9) | 22 (18.5) | 485 (21.0) | 0.51 |
| Ro |  | 756 (31.1) | 33 (27.7) | 723 (31.3) | 0.41 |
| La |  | 313 (12.9) | 8 (6.7) | 305 (13.2) | 0.039 |
| Lupus anticoagulant |  | 615 (25.3) | 31 (26.1) | 584 (25.3) | 0.84 |
| Anticardiolipin |  | 1118 (46.0) | 72 (60.5) | 1046 (45.2) | 0.001 |
| Beta 2 glycoprotein 1 |  | 446 (18.3) | 21 (17.6) | 425 (18.4) | 0.83 |

All values represented are numbers (percentage), unless otherwise specified.

*p-value for comparison between cancer and no cancer groups. When adjusting for multiple comparisons, p≤0.005 is statistically significant.

**Supplemental Table 3. Drug exposure (ever use) by cancer status.**

| **Drug** |  | **Overall (N=2431)** | **Cancer (N=119)** | **No cancer (N=2312)** | **p-value*** |
| --- | --- | --- | --- | --- | --- |
| Hydroxychloroquine |  | 619 (25.5) | 27 (22.7) | 592 (25.6) | 0.48 |
| Mycophenolate mofetil |  | 2127 (87.5) | 107 (89.9) | 2020 (87.4) | 0.42 |
| Azathioprine |  | 640 (26.3) | 38 (31.9) | 602 (26.0) | 0.15 |
| Cyclophosphamide |  | 451 (18.6) | 26 (21.8) | 425 (18.4) | 0.35 |
| Belimumab |  | 29 (1.2) | 0 (0.0) | 29 (1.3) | 0.21 |
| Rituximab |  | 84 (3.5) | 5 (4.2) | 79 (3.4) | 0.64 |
| Prednisone |  | 2082 (85.6) | 101 (84.9) | 1981 (85.7) | 0.81 |
| Premarin |  | 332 (13.7) | 26 (21.8) | 306 (13.2) | 0.008 |
| Estrogen-containing oral contraceptives |  | 1325 (54.5) | 72 (60.5) | 1253 (54.2) | 0.18 |

All values represented are numbers (percentage), unless otherwise specified.

*p-value for comparison between cancer and no cancer groups. When adjusting for multiple comparisons, p≤0.006 is considered statistically significant.

**Supplemental Table 4. Cancer sites observed in the Hopkins Lupus Cohort (N=119 cancer cases).**

| **Site** | **Number** | **% of cancers after cohort entry** |
| --- | --- | --- |
| All | 119 |  |
| **Female cancers** |  |  |
| Breast | 22 | 18.5 |
| Uterus | 2 | 1.68 |
| Ovary | 0 | 0.00 |
| Fallopian tube | 0 | 0.00 |
| Cervix | 11 | 9.24 |
| Vagina/vulva | 13 | 10.9 |
| **Male cancers** |  |  |
| Prostate | 0 | 0.00 |
| **Other cancers** |  |  |
| Hematologic | 10 | 8.40 |
| Multiple myeloma | 2 | 1.68 |
| Salivary | 1 | 0.84 |
| Lung | 11 | 9.24 |
| Stomach | 2 | 1.68 |
| Duodenum and other small bowel | 2 | 1.68 |
| Colorectal | 10 | 8.40 |
| Anus | 2 | 1.68 |
| Pancreas | 2 | 1.68 |
| Liver | 3 | 2.52 |
| Thyroid | 9 | 7.56 |
| Melanoma | 8 | 6.72 |
| Kidney | 4 | 3.36 |
| Bladder | 3 | 2.52 |
| Brain | 1 | 0.84 |
| Unknown primary | 1 | 0.84 |

Totals may not add to 100% due to rounding.
